# IMPACT OF COGNITIVE RESERVE IN OBJECTIVE AND SUBJECTIVE SUCCESSFUL AGING

**DOI:** 10.1093/geroni/igac059.2163

**Published:** 2022-12-20

**Authors:** Neyda Ma Mendoza Ruvalcaba, Elva Dolores Arias-Merino, Maria Elena Flores-Villavicencio, Melina Rodríguez Díaz

**Affiliations:** University of Guadalajara, Guadalajara, Jalisco, Mexico; University of Guadalajara, Guadalajara, Jalisco, Mexico; University of Guadalajara, Guadalajara, Jalisco, Mexico; University of Guadalajara, Guadalajara, Jalisco, Mexico

## Abstract

Cognitive reserve (CR) refers to the capacity of the brain to withstand changes due to age or disease-related pathology, showing how flexibly and efficiently the individual makes use of available brain resources. This study aims to explore the contributions of the CR to understanding successful aging (SA).(Project-Conacyt-256589)Population based, random sample included n=656 community-dwelling older adults 60-years and older (mean age=72.8, SD=7.6 years, 58% women). CR was measured by their main indicators: education, life-long learning, being bilingual, participation, use of information and communications technology. Objective SA was operationalized as no important disease, no disability, physical functioning, cognitive functioning, and being actively engaged. Subjective was an appreciation if they considered themselves as successful agers. Sociodemographic and health data were also asked. Pearson′s correlation test and MANOVAs were performed.In total 11.2% met the criteria for SA, although 76% considered themselves as successful agers. CR was significantly related to subjective and objective criteria of SA(p<.000), except to no-important diseases. CR explains in general 20% of the variance in objective SA, specifically explains 28% of variance in the criteria of high cognitive function, 18% of the variance in disability, 11.3% of life engagement, 8% of physical functioning, and 2% of disease-free criteria. Also, CR explains 10% of the variance in subjective SA. This study has shown that CR the is related to SA, this set possible targets for cognitive interventions to promote resiliency of the brain not only for preventing cognitive pathologies, but also for encouraging successful and healthy aging in older adults.

